# ‘All of a sudden, you know, you can’t go to these services, because of the risk of infection’: Audiological service considerations at residential care homes for older persons during COVID-19

**DOI:** 10.4102/sajcd.v69i2.904

**Published:** 2022-08-10

**Authors:** Victor de Andrade, Rethabile R.M. Landman

**Affiliations:** 1 Department of Speech Pathology and Audiology, School of Human and Community Development, University of the Witwatersrand, Johannesburg, South Africa; 2 Department of Speech Pathology and Audiology, Faculty of Humanities, University of the Witwatersrand, Johannesburg, South Africa

**Keywords:** residential care homes for older adults, audiological services, COVID-19, hearing loss, South Africa

## Abstract

**Background:**

Residential care homes for older persons were especially affected during the coronavirus disease 2019 (COVID-19) pandemic lockdowns which resulted in limited social interactions and service provision. Communication became challenging due to the prophylactic use of masks and social distancing.

**Objectives:**

This qualitative research study set out to explore audiological service considerations in residential care homes for older persons during the COVID-19 restrictions.

**Method:**

Through purposive sampling, nine managers from residential care homes for older persons in Johannesburg participated in semi-structured, online interviews. The transcriptions of these recorded interviews underwent thematic analysis.

**Results:**

Managers employed various strategies to attend to residents’ audiological needs, audiological health, hearing aid use, and hearing aid provision. Furthermore, it transpired that other health related services were prioritised over audiological services in general, but especially during the pandemic lockdowns. Managers reported that staff had to use various communication strategies due to COVID-19 precautions and that masks and social distancing made communication more challenging for residents with hearing loss. Moreover, isolation and modified service provision were extremely taxing on residents.

**Conclusion:**

This study highlights the need for continued audiological services at residential care homes, but also the need to balance audiological needs with other health needs because these seem to be prioritised over hearing loss, especially in this population who may have limited agency and choice in the health care options available to them. Furthermore, adapted strategies need to be considered to support communication considering COVID-19 precautions so that communicative difficulties do not exacerbate lockdown isolation.

## Background

### Residential care homes for older persons in South Africa

Manderson, Harling and Withem (2019) argue that, in South Africa, care facilities such as residential care homes for older persons are usually a last resort for the older persons. Nonetheless, it is estimated that there are approximately 1150 residential care homes for older persons in South Africa, of which only 415 are registered with the Department of Social Development (Mahomedy, 2017). Perold and Muller (2000) suggest that residential care homes for older persons:

[*C*]ame into existence in South Africa within the social work domain because of various social problems including loneliness, economic and housing problems, deteriorating mobility of the older persons and lack of family and other support systems for them in the community. (p. 1)

Broadly, Manderson et al. (2019) state that care for the elderly usually includes assisting with activities such as, bathing, getting dressed, eating, going to the toilet, among others. However, Manderson et al. (2019) question whether these activities are enough to allow elderly persons to participate in society and that there are considerable shortcomings and a lack of formal care when it comes to caring for the elderly in South Africa. Lloyd-Sherlock (2019) adds that the legacy of Apartheid permeates into residential care homes for older people where older white people may have access to them while older black people may be cared for by their families.

Mahomedy (2021) predicts that the demand for care among older persons will escalate and that South Africa is not prepared for this increasing demand. As stated earlier, because many homes are not registered with the Department of Social Development, this may suggest that these homes do not comply with the *Older Persons Act,* No. 13 of 2006, Section 18, subsection 1a, which states that no one should operate a residential facility for the elderly unless it has been registered (Mahomedy, 2017). Furthermore, there is a shortage of trained staff who have the skills to take care of the elderly (Mahomedy, 2017, 2021) which also raises questions about the training of the staff at residential care homes for older persons regarding audiological services, especially because staff at residential care homes for older persons may have very little knowledge about hearing services, especially hearing aids (Pryce & Gooberman-Hill, 2012).

Finances appear stretched because the high levels of poverty and unemployment in the country add to the pressures on older persons and their children who support them financially (Kang’ethe, 2018). The government’s social grant for older persons as well as the subsidies to homes are not enough to cover the costs of running the homes (Mahomedy, 2021). Although homes have tried to explore other fundraising to cope with the financial demands, the coronavirus disease 2019 (COVID-19) pandemic interrupted such fundraising and resulted in more deficits and closures. (Mahomedy, 2021). Therefore, considering these financial constraints, although hearing amplification devices such as hearing aids are recommended for people with presbycusis (Sprinzl & Riechelmann, 2010), Sooful, Dijk and Avenant (2009) found that hearing loss in South Africa is considered a low priority because hearing amplification technologies are expensive for most South Africans and people do not have money for transport to go for repairs and retubing, nor the money to buy hearing aid batteries (Sooful et al., 2009).

### Hearing and older persons in residential care homes

Residential care homes for older persons may not always be able to offer favourable communication environments for persons with a hearing loss. For example, residents in Pryce and Gooberman-Hill’s (2012) study stated that they were unable to cope with background noise of music, television, other residents’ conversations, or the noise of crockery.

Boi et al. (2012) argue that hearing aids can improve the quality of life in clients who use them regularly and correctly and that a decline in depressive symptoms was noted, the burden on caregivers lessened, feelings of social isolation also declined in the participants of this study after they were fitted with hearing aids. However, in South Africa, older persons do not seem to use hearing aids regularly and correctly (Moroe & Vazzana, 2019) which, consequently, could result in social isolation and diminished quality of life. Furthermore, untreated hearing loss has been associated with incident dementia because of prolonged auditory deprivation, feelings of social isolation, and feelings of being unfamiliar with the environment (Lin et al., 2011).

### COVID-19 and the hearing of older persons in residential care homes

Due to COVID-19, precautionary measures had to be taken to limit the spread of the virus, including social distancing, isolation, and the use of masks. Dipa (2020) found that older persons in residential care homes in Gauteng, South Africa, felt lonely and depressed during the national lockdown of March 2020. Families of residents were not allowed to visit, and residents were confined to their rooms with very little social interaction while also having to adapt to using smartphones and social media applications to communicate with their families.

The American Speech-Language-Hearing Association (ASHA, 2020) states that masks muffle speech, making it difficult to hear and understand speech, especially for those with hearing difficulties. Specifically, masks degrade speech because they act as low pass filters and attenuate high frequencies from 2000 Hz to 7000 Hz by between 4dB and 12dB, making it very difficult for people to hear, especially people who already have a hearing loss (Goldin, Weinstein, & Shiman, 2020). To exacerbate matters, Goldin et al. (2020) point out that when masks are used in environments with background noise and the absence of visual cues from facial expressions, speech is mostly perceived as unintelligible for many people.

Transparent masks have been offered as a potential opportunity to allow for lip-reading while still offering a barrier to mitigate for COVID-19 spread (Chodosh, Weinstein, & Blustein, 2020; Correia & Ferreira, 2021). Despite the potential benefit, Chodosh et al. (2020) point out that there are few manufacturers of these masks and, therefore, Correia and Ferreria (2021) advocate for more investment in transparent masks. However, as Thibodeau, Thibodeau-Nielsen, Tran and Jacob (2021) point out, transparent masks can become fogged up by the warm breath on the inside of the mask, thereby obstructing the view of the speaker’s mouth. Nonetheless, Thibodeau et al. (2021) offer suggestions to minimise the fog and these suggestions include ‘simply applying a thin film of a liquid soap or bubbles to more expensive options of a product designed for antifog’ (p. 780).

### Rationale of the study

Therefore, because older persons may be more susceptible to presbycusis and since hearing loss in older adults may result in isolation, reduced quality of life, communication challenges, depression, and dementia, it was deemed necessary to explore audiological service provision in residential care homes during a time when the focus was on mitigating for COVID-19. Furthermore, because audiological services may not have prominence (Joubert, Sebothoma, & Kgare, 2017), these audiological services may have been relegated due to other competing demands. This study, therefore, sought to provide a description of the audiological service considerations at residential care homes for older persons in Johannesburg during COVID-19 restrictions in South Africa.

## Methodology

A qualitative research design was employed to provide a description of the audiological service considerations at residential care homes for older persons in Johannesburg during COVID-19 restrictions by interviewing the managers of the homes.

Through purposive sampling, the managers of 30 residential care homes were invited, via phone calls and emails, to participate in the research project and nine agreed to participate. Some of the managers who declined participation cited competing responsibilities such as COVID-19 demands and the first rollout of vaccinations which was happening at the time of data collection. Nine interviews were conducted, two of which were joint interviews while one participant was involved at two homes. Table 1 presents information about the participants.

**TABLE 1:** Participants’ profile.

Participant number	Status of residential care home	Participant designation
P1	Non-profit organisation	Nursing services manager
P2.1	Privately owned	P2.1 – Manager
P2.2		P2.2 – Matron
P3	Subsidised by government and dependent on donations	Nursing services manager
P4	Privately owned	Nursing services manager
P5	Privately owned	Manager
P6.1	Subsidised by government and dependent on donations	P6.1 – Manager
P6.2		P6.2 – Deputy manager
P7 (mainly based at the privately owned home but does outreach programmes once a month at a government home)	Privately owned Subsidised by government and dependent on donations	Nursing services coordinator
P8	Non-profit organisation	Nursing manager
P9	Non-profit organisation	Resident social worker

Semi-structured interviews were conducted utilising an interview guide informed by the aim of the project and a review of the literature. The interviews were conducted between 25 May 2021 and 21 June 2021 when South Africa was struggling with the Delta variant of COVID-19 and went from adjusted level one lockdown regulations to level four lockdown. These semi-structured interviews were conducted using WhatsApp video call, Zoom, and Microsoft Teams Meetings. The interviews which were conducted on Zoom and Microsoft Teams were successfully recorded using the recording options on the applications while the interviews from the WhatsApp video calls were recorded on a secondary cellular phone. The interviews were conducted in participants’ preferred languages where code switching also occurred.

The recordings were used to transcribe the interviews and where different languages were spoken, the transcriptions reflected those. For example, P1 preferred to speak a mixture of Setswana and English and then the one author, R.R.M.L., transcribed the interview into English and Setswana. After transcription, member checking was conducted by emailing transcriptions of the interviews to the participants so that they could comment on the accuracy of the transcriptions.

Thereafter, the transcriptions were analysed using thematic analysis. Braun and Clarke (2008) suggest flexibility without focussing on preconceived themes to welcome themes that arise in the data and therefore deductive and inductive approaches were employed.

### Ethical considerations

Prior to conducting the interviews and after obtaining clearance from the University of the Witwatersrand Human Research Ethics Committee (STA-2021-26), a pilot study was conducted. Thereafter, letters of permission were sent to homes seeking permission from the management committee or board of the homes to interview the managers. Participant information sheets were sent to the managers of the homes which had granted permission for participation and informed consent was received from them prior to commencement of the interviews.

## Results

The data analysis revealed that COVID-19 affected communication and audiological service provision for older persons in residential care homes. Seven themes emerged from the data analysis, namely, (1) audiological services were interrupted due to COVID-19, (2) other health services were prioritised over audiological services due to COVID-19, (3) COVID-19 mask-wearing adversely impacted on communication with older persons in residential care homes, (4) communication had to be adjusted for residents who have hearing loss during COVID-19 restrictions, (5) COVID-19 increased the sense of isolation for residents, (6) financial constraints limited the provision of audiological services, and (7) COVID-19 has impacted on the confidence in hospital attendance for audiological service provision. These themes are detailed hereafter.

### Audiological services were interrupted due to COVID-19

Due to lockdowns and restricted access, there were limited opportunities for external audiological services as per P5 (female, manager).

‘Pre-COVID, once a month she’d [*audiologist*] come for general ear health checks and then make her recommendations thereafter. Now, … uhm eight months maybe, six to eight months, I mean there was one time in between, you know, since the start of the pandemic.’

Because of these interruptions, care staff at residential care homes were not able to continue offering services usually offered by audiologists. For example, P4 (female, nursing services manager) expressed that even though the audiologist had shown her and the staff how to take care of the hearing aids, she is the only one who helps residents with the hearing aids:

‘You know, these things are so small and so finicky, and uh you know, temperamental so if you put the batteries in the wrong side, up. You, you know, you might break, break the inside. We’ve had that with somebody tried to force something in there that shouldn’t be done. So normally when it’s you know, in pieces or that doesn’t want to work, or we’ve got a high pitch tone or something. The staff will bring it to me and say, there’s a problem here. So, it’s a box on my, on my table and they’ll leave it with a note to say whose is this, whose this is and then I just manage it from there ….’

The strict lockdown restrictions resulted in limited audiological service provision, especially because there were audiology practices and services which were closed during the stricter levels of lockdown. However, it seems as though the availability of audiological services diminished due to COVID-19 as per this excerpt from P4 (female, nursing services manager):

‘Quite bad, like I said, it’s like the industries uhm took a strain. I think most people became aware of the needs, the companies were, you know, because people were let off, uhm, companies were closed, couple of places that I phoned that told me they don’t have staff to send out to us. You know, you can see, it’s taken a knock and the availability shrunk.’

Furthermore, access to audiological services was limited, even those which were still available during the COVID-19 restrictions as P4 (female, nursing services manager) reported: ‘Uh, yes, transport, getting there, we’re not allowed to transport people up and down, unless the audiologist can come to them…’. P7 (female, nursing services coordinator) offered strategies that were employed to address transport service interruptions:

‘But the problem with Home R is, obviously with the transport, buses have been cut to get there, but now they know about Uber and Taxify, they’re fine, and then they help each other out to get to the appointment ….’

Participants spoke of how residents’ attendance for audiological service provision became risky and therefore had to stop. P7 (female, nursing services coordinator), spoke of how having to go to an audiologist suddenly became a risk:

‘[… *B*]ecause all of a sudden, you know, you can’t go to these services, because of the risk of infection, you see, so it has, the fact that you just cannot, as an elderly person if you’re not vaccinated or fully vaccinated, you cannot go because there’s a risk when getting to these services, the access has been now blocked in a way….’

### Other health services were prioritised over audiological services due to COVID-19

In addition to COVID-19 health concerns, managers reported that residents had a variety of health concerns, including Turner’s syndrome, diabetes, arthritis, hypertension and hypotension, stroke, Parkinson’s disease, dementia, and Huntington’s disease, which also needed to be balanced with other needs. The findings from this study revealed that survival strategies were prioritised during the pandemic. Participants spoke of the need to focus on life threatening aspects as exemplified by P4 (female, nursing services manager) when she said:

‘Sjoe, uhm it all goes as to what’s the basic need at that present moment. I first go with what’s the most important thing, if it’s life threatening or threatening a resident’s, social ability or inability. If it threatens somebody is able to you know, being to have a normal day for me that’s a priority, then I start there. Uhm, you know, uh if it’s just something smaller, like a resident tells me I’ve got one hearing aid, but the other one is a little bit, you know, that one, I’ll leave a little bit later and just manage the one without the hearing aid first….’

P9 (female, resident social worker) stressed the prioritisation during COVID-19 when she said: ‘So services are always prioritized in terms of medical and nursing and physical, emotional care…’ and P3 (female, nursing services manager) reinforced this notion of the relegation of audiological services ‘The audio and the speech cases, they are also important but now the focus is on the COVID side and the and medical side, you see …’. Even though participants expressed that other health concerns take priority, it seems as though dementia coupled with hearing loss has devastating effects on the elderly as per P5 (female, manager):

‘… I would say about 80% of them have hearing aids and use hearing aids. Without them, they can’t really function…management of their dementia and Alzheimer’s and anxiety. So, from a mental health perspective, that definitely takes absolute priority….’

and P8 (female, nursing manager):

‘[…*T*]hat is difficult because you have a combination, oftentimes in our home where, it is the natural decline of the body, so the residents feel like, I’m aging, and I have to accept it. Plus, I have hearing loss, and oftentimes early onset dementia, which creates almost the perfect storm.’

Participants referred to the limited provision of audiological services due to insufficient information and prominence of audiology, especially during a time when COVID-19 was predominant. P9 (female, resident social worker) referred to the invisibility of audiology when she said:

‘… Audiology is a field that is, honestly very fuzzy for us at the moment … I think there’s a lack of knowledge about audiology, about its delineation, the boundaries, the servicing, when it’s required, what to look for, I think a lot of education is required in most facilities., around, audiology, it’s about advocating, you know.’

P4 (female, nursing services manager) reinforced this idea when she said:

‘Uhm, you know what, I think if maybe, if there’s maybe a little bit more pamphlets and things that’s rather a little bit more advertising, uhm making themselves visible to us….’

Consequently, due to the reduced prominence, audiological services were not always foregrounded as P1 (female, nursing services manager) pointed out:

‘Well, the thing is we never say that we are going out to check the hearing status of our patients. Hore okay ha re kopeng doctor [*That let’s ask the doctor*] … Lets check the ears of our residents and those types of things. Errr to be honest, I don’t think … er … much as we know that progressive hearing loss is something that affects the elderly … It’s not something that we say … er guys … this week a ke re check’eng [*let’s check*] the hearing capacity of our residents … because we have noticed that Nkgono Y and Nkgono X have got a problem when we speak to them.’

### COVID-19 mask-wearing adversely impacted on communication with older persons in residential care homes

Participants reported that COVID-19 mask-wearing protocols made communication difficult. For example, P1 (female, nursing services manager) said:

‘It is a problem for them to keep their masks in situ besides it’s very difficult for a person, gore o moutlwehoreontsareng ha a kentse mask [*for a person to hear what you are saying when you are wearing a*] mask….’

P2.1 (female, manager) reported that ‘The mask, the mask, muffles your voice somehow’. P3 (female, nursing services manager) stated that sometimes carers had to take off their masks for residents to be able to understand them:

‘Yoh, they can’t hear because now with a mask and the residents have got the masks, and the staff have got masks on, it’s so difficult to communicate, you know. So, at some stages we just take the mask off, I mean, what can we do, really?’

### Communication had to be adjusted for residents who have hearing loss during COVID-19 restrictions

Coronavirus disease 2019 (COVID-19) containment strategies have resulted in communication challenges for residents. Participants said that prior to COVID-19 mask-wearing obligations, face-to-face communication was reported to be very helpful, especially because then residents could lip-read. Subsequently, due to mask-wearing, participants reported that carers have had to resort to speaking more loudly. Despite the potential benefit of carers speaking more loudly, the participants said that this louder speech is not always ideal as it does not promote discretion as per P1 (female, nursing services manager) in this excerpt:

‘Jah, jah like, we do talk loud buuut, but, you can’t do, you can’t do loud, you know. Like for instance, when you’ve got an incontinent person not to embarrass them. You want them not to get off the chair and tell them to go with you to the room, so I’ll just tell them come please with us to the room, but you can’t you say, you wet the chair please follow me at your room, you know, then aaall of Home N knows that chair is now wet, you know, so you need to be discreet … Uhm jah, and sensitive. Get the resident up, make sure nobody notices there’s been a glipsie, and then get the chair out of the equation and get the chair sorted and replace another chair there without anybody noticing, and then take the resident to be cleaned up, and you know not to make this you know like a whole big shpiel.’

Due to these challenges, participants said that carers have had to find alternative ways of communicating with residents. For example, P9 (female, resident social worker) spoke of written and diagrammatic strategies when she said: ‘… as well as writing and drawing where possible. So, most residents, uhm, the problem is that it takes time and effort…’. P7 (female, nursing services coordinator) stated that carers use hand gestures, as depicted in the following excerpt, ‘…whether you do certain hand gestures with them, you find a way. Like, I said, they’re creative in a way, they always find a way…’.

Furthermore, due to limitations placed on social interaction and visitors, P3 (female, nursing services manager) added that residential care homes were quieter during COVID-19 restrictions: ‘…because there’s not too much noise with us here, it’s always quiet and uhm, it’s always quiet and we don’t have any social gatherings at the moment…’.

### COVID-19 increased the sense of isolation for residents

Participants spoke of the isolation of residents due to COVID-19, especially with protocols relating to self-isolating and quarantine. P9 (female, resident social worker) explained:

‘So, when, when, especially during the first wave, we had that we couldn’t even access the wards, and people were extremely isolated, they were. There were a few weeks where people were locked in their rooms, where they couldn’t even go out. So that was detrimental, it’s devastating, you know. You weigh up the physical safety, and you weigh everything else and nothing. I mean to do that to someone that feels cruel, we could see people withering, people were withering from lack of being able to socialize….’

Concomitant challenges seemed to aggravate the already difficult situation as highlighted by P1 (female, nursing services manager) who spoke of how some residents, especially those who had dementia, struggled to adhere to the COVID-19 protocols:

‘When it was obvious now that we had to go to level 5, we had to have isolation rooms and quarantine rooms, but it was very difficult because some of the patients, waitsi [*you know*] older people especially banaleng [*that have*] dementia o re o mokentse [*you will send to*] into isolation or quarantine o tlatswa [*then they will go out*], o tlainsist’ahorenikslenna [*they will insist that they*] want to sit with the other mogolos [*residents*].’

In order to alleviate isolation, P7 (female, nursing services coordinator) reported that they were able to make use of technology and virtual platforms such as WhatsApp video call to facilitate communication between residents and their families and friends outside the residential care homes, as seen in the following excerpt:

‘[… *B*]ut social media, you know, social media like these platforms, helped a lot, we have assisted a lot of residents to get connected especially with WhatsApp, so you can communicate with them.’

### Financial constraints limited the provision of audiological services

Stretched financial resources, especially in a time of competing demands due to COVID-19, seemed to impact on audiological services. P1 (female, nursing services manager) said:

‘Other institutions, tsona [they] can afford to have a full-time audiologist. At least maybe rona [we] we can use the services offered by the final year medical, I mean audio students to come and look at the hearing of our residents because we handle very poor and the low of the lowest socio-economic group, waitsi [you know]….’

P3 (female, nursing services manager) described the absence of services because of financial constraints when she said, ‘… Jah no we don’t have those services. You know, our home is a subsidized by the government, so we don’t have all those resources here…’. It also seems as though private medical insurance is not a guarantee that audiological services can be provided as exemplified by P3 (female, nursing services manager) who said:

‘[… *A*]nd even for the people on medical aids, and the, the funds available for audiology is not sufficient enough, so finances definitely plays a massive role to the residents, whether they’re for medical aid or not….’

Because of the redirecting of funds due to COVID-19, hearing aid services were dependent on the availability of funds at that time as per P8 (female, nursing manager) who said that if residents need hearing aids but do not have funds to buy hearing aids, then they are referred to the local public hospital but that audiological services as not guaranteed, ‘Jah, that will be literally dependent on whatever is happening in the government.’ Also, because fundraising was limited due to COVID-19, money that could have been used for hearing aids is not available as per the excerpt from the interview with P4 (female, nursing services manager): ‘Uhm, we’ve got a couple of people that’s on the Benevolent Fund, but, you know, everyone has taken some strain’. Consequently, older persons with hearing loss have had to endure communication difficulties as per P3 (female, nursing services manager): ‘Sometimes they don’t, sometimes we don’t have money to buy the batteries. So, they will stay without, jah, they can’t hear anybody talking, and all that…’.

### COVID-19 has impacted on the confidence in hospital attendance for audiological service provision

Residents seemed reluctant to go for audiological services at hospitals as exemplified by P8 (female, nursing manager):

‘I think the residents are more sceptical, about going to actually the clinic for anything, because they don’t want to go into isolation … As soon as it comes to the government facilities, there’s no social distancing, there’s no systems in place … they are also afraid that they might get exposed to the virus there … fear of either contracting COVID or having to go into isolation.’

Furthermore, the waiting times at hospitals increased residents’ reluctance as per the example offered by P3 (female, nursing services manager):

‘You know, the other thing, they don’t want to go to hospitals because they stay there for a long time. That’s why, that’s one of the things that they make, them not want to go. So, they think, I’m gonna go there and I’m gonna stay for the whole day there and all that so it’s better, it’s better to leave it. I’m not going.’

Moreover, as managers, they themselves feared that residents would contract the virus at crowded hospitals, as per P7 (female, nursing services coordinator):

‘[… *B*]ut now (name) Hospital is also full, and I don’t want them to go there, to be honest with you, I don’t want them going there and catching something or getting anything.’

Participants therefore spoke of strategies which they adopted to address this interruption in audiological service provision. For example, P6.1 (female, manager) stated that they made arrangements for the hearing aids to be sent to the audiologist or for the audiologist to come in, be screened, and observe all the COVID-19 protocols so as to provide the necessary audiological services:

‘…So, what we do is we do have, we either send the, the hearing aid to the audiologist and they then clean it and look at it, okay, but we have allowed some of the audiologists in and they have to then dress in, you know, their masks, visors and put their gloves on….’

## Discussion

This qualitative study has offered insights into audiological service considerations at residential care homes for older persons in Johannesburg during COVID-19 restrictions in South Africa. The results of this study showed that pre-COVID-19 challenges in South African residential care homes, including a shortage of resources and equipment (Maphumulo & Bhengu, 2019) as well as poor audiological services (Sooful et al., 2009) were amplified during the COVID-19 crisis.

Participants reported that COVID-19 precautions such as mask-wearing had a negative impact on communication because residents struggle to hear them. This finding is supported by Goldin et al. (2020) who found that a simple medical mask reduced the sound by 3dB to 4dB, while the N95 medical masks reduce the sound by 12dB, a sentiment shared by participants who, for example, said that the mask ‘muffles’ their voices which makes it very difficult for residents to hear them. The results also showed that sometimes staff at old age homes had to become noncompliant with the COVID-19 precautions because, even though it is in everybody’s best interests to comply with the COVID-19 regulations, they do sometimes pull down their mask to communicate effectively with residents because, with their masks on, residents struggle to hear them.

Participants spoke of communication strategies that had to be adopted when communicating with persons who have hearing loss during COVID-19. The results revealed that staff speak louder to residents who have hearing loss, but participants also said that speaking louder could compromise residents’ privacy or cause embarrassment for residents, as per the example offered earlier relating to a resident who had urinated. Similarly, Newton and Shah (2013) propose the need to speak clearly and not yell when communicating with persons who have a hearing loss because that might infringe on people’s privacy because others will hear what is being said. In addition to enhanced spoken communicative strategies, participants reported using other communication strategies which echo other literature where, for example, Newton and Shah (2013), encourage the use of pictures, cards, and writing down information. Gestures and pointing were also mentioned by participants and these modes of communication are also encouraged by Newton and Shah (2013) when communicating with persons who have hearing loss.

From the results of this study, it seems as though other services are prioritised over audiological services. It could be said that hearing loss is an invisible disability (Mackenzie & Smith, 2009) and even before the declaration of the pandemic, in our context, it is reported that hearing loss was not prioritised because of the burden of disease and the allocation of resources to, for example, human immunodeficiency virus (HIV), acquired immunodeficiency syndrome (AIDS), tuberculosis (TB), and malaria (Sooful et al., 2009). Furthermore, participants in this study said that audiological services did not have the same prominence as other services. Joubert et al. (2017) state that there is a serious lack in the public awareness of audiology, regarding the profession itself and services offered by audiologists. The results confirmed this assertion at residential care homes, and it appears necessary to follow Joubert et al.’s (2017) suggestion for collaboration between audiologists, universities, and the National Department of Health to implement strategies that will increase knowledge and awareness about audiology and audiological services.

Notwithstanding the prioritisation of COVID-19 needs, the need for audiological services remained, especially because of the possible audiological sequelae of COVID-19 (Almufarrij & Munro, 2021) but also because benefits associated with auditory stimuli, sound recognition, and sound discrimination as well as the social benefit and emotional well-being (Noble & Gatehouse, 2006) for people with hearing loss. Dawes et al. (2015) point out that hearing loss can exacerbate dementia in the elderly and that addressing hearing loss can improve cognitive performance in the elderly. As evidenced in our study, when audiologists were not able to offer services during the interruption, residents could not benefit from their hearing aids and, therefore, could experience cognitive sequelae. This points to the audiological benefits of audiological services which ought to be considered a crucial service.

Moreover, participants spoke about the isolation experienced by residents at homes during COVID-19. Older persons who have hearing loss are already at risk of social isolation, social withdrawal, and poor quality of life (D’Haese et al., 2019); thus, this current study showed that residents’ isolation due to COVID-19 restrictions compounded their isolation. Participants spoke of strategies such as using WhatsApp video calls for residents to maintain contact between them and their families outside. It is proposed that through phone calls, social media, and the internet, residents can maintain contact with family and friends, especially to mitigate for ‘the mental health problems of the elderly caused by COVID-19’ (Lee, Jeong, & Yim, 2020, p. 3).

The participants in this study spoke of the need for audiologists to offer audiological services at residential care homes because staff at the homes struggled to do so. In addition to limited access to health care, including audiological services and a lack of proper equipment used in audiology, there is a limited number of health professionals that can attend to matters pertaining to hearing loss (Harris & Dodson, 2017). Also, as reported by participants in this study, there has been a reduction in audiological services because of COVID-19. Pillay, Tiwari, Kathard and Chikte’s (2020) study found that there was already a shortage of audiologists in the workforce in South Africa pre-COVID-19, so this study reinforces the need for increased audiological service provision in our country due to the workforce consequences of the pandemic.

Financial challenges pervaded the results of this study. Maphumulo and Bhengu (2019) reinforce that the healthcare system in South Africa has had problems since Apartheid where the distribution of resources was unequal based on race and geographical location, especially prejudicing poorer and black communities and that, in general, there was a shortage of resources and shortage of skilled professionals. Mhlanga and Garidzirai (2020) further argue that even though South Africa is almost 30 years into its full democracy, there is still inequality in the health sector. The findings of this study found that older persons were reluctant to attend health facilities due to concerns about COVID-19 and, according to Mhlanga and Garidzirai (2020), approximately 70% of South Africans depend on the public health system which suggests that many older persons who would otherwise be accessing these services, could not, or were reluctant to because of concerns about COVID-19 safety at hospitals. Even pre-COVID-19, Maphumulo and Bengu (2019) found that there are several challenges such as poor hygiene and poor infection control measures at hospitals and these concerns seem to have extended to COVID-19 matters. This current study suggests the need to attend to these concerns while also looking at improving service delivery to improve residents’ experiences of visiting health facilities.

## Conclusion

In conclusion, this research study has revealed that COVID-19 restrictions have had a detrimental effect on audiological services, as well as communication, for people who live in residential care homes for older persons. Managers reported on the effect of the restrictions as well as strategies to try address these challenges. It has been very difficult for staff at residential care homes to balance the life-sustaining and COVID-19 protection needs with residents’ audiological service needs. Furthermore, interruptions to an already strained audiological service, workforce challenges, as well as financial constraints are among the concerns. Understandably, COVID-19 containment took priority, but this study revealed the particular audiological challenges posed by the restrictions. Furthermore, the study highlighted that, although audiological services may not get the same priority, the communicative challenges faced by older persons who have hearing loss can increase loneliness and isolation, mental health challenges, and cognitive health issues. The COVID-19 pandemic appears to have added a level of complexity to the managers’ attendance to audiological needs.

If services are interrupted, to maintain communication, enhanced communication strategies need to be included, for example, to compensate for mask-wearing. To account for masks, Saunders, Jackson and Visram (2021) suggest transparent masks to facilitate lip-reading while Thibodeau et al. (2021) provide options for transparent masks that do not fog up. Furthermore, suggestions and guidelines have been provided on how audiologists can modify hearing aid programme settings and frequency responses to account for the specific frequencies which are degraded due to mask-wearing (Saunders et al., 2021). Furthermore, Schlögl and Jones (2020) suggest that when speaking to older persons during COVID-19 restrictions, especially when wearing masks, it is important to attend mindfully when interacting with older persons and to maintain calm, despite communication frustrations and breakdowns. They specifically mention approaching older persons from the front so that, besides enhanced awareness and communication, it also shows respect for the older person by not catching them unaware. Furthermore, for staff working with older persons at residential care homes, their advice for communicating with older persons during these COVID-19 restrictions could be beneficial:

[*A*]void noise and overwhelming stimulus and make surethe older person is wearing glasses or hearing aids, ifneeded, then slowly communicate one point at a time. Useshort, simple sentences and underline your words with gestures.Make your statement or ask your question and thenpause. Keep your voice even, tone gentle, and speech slow. (Schlögl & Jones, 2020, p. E12)

Cognisant of the health and life-threatening aspects of COVID-19, audiological services ought to continue as essential services with all the necessary precautions to reduce the sensory deprivation, cognitive risk, and socio-emotional difficulties which may arise from the combination of COVID-19 restrictions and hearing loss. Senescence may have effects on the whole body and, therefore, specific strategies need to be adopted at residential care homes to offer holistic care and a balanced service provision that includes audiology, even when there are competing needs as evidenced during the COVID-19 restrictions, so that older residents can benefit from a well-rounded service provision.
